# Management of cloacal exstrophy: Experience from tertiary hospital, Tanzania. Case series

**DOI:** 10.1016/j.ijscr.2024.110387

**Published:** 2024-09-30

**Authors:** Geofrey P. Chiloleti, Gabriel Mtaturu, Sirili Harya, Herry G. Kibona, Boniface Kilangi, Fransia A. Mushi

**Affiliations:** a Department of Surgery, School of Medicine, Muhimbili University of Health and Allied Sciences, Dar es Salaam, Tanzania; b Department of Urology, Muhimbili National Hospital, Dar es salaam, Tanzania

**Keywords:** Case series, Cloacal exstrophy, Bladder exstrophy, Omphalocele, Elephantoid trunk, Cecal fistula

## Abstract

**Introduction and importance:**

Cloacal exstrophy (CE) is defined as a complex anomaly that affects the urogenital and intestinal tracts. It is the most serious form of anomaly that is described within the so-called exstrophy-epispadias complex. These malformations usually present a challenge in the management of particular conditions, as most of these forms require multiple surgeries, resulting in the use of multidisciplinary approaches, including reconstructive urologists, pediatric surgeons, orthopedic surgeons, endocrinologists, pediatricians, psychologists and nutritionists. Additionally, these patients present with ambiguous genitalia, which is another aspect that needs to be taken into consideration during the management of this condition.

**Case presentation:**

The first patient, a baby who was 8 days of life and referred from a peripheral hospital, presented with classic features of cloaca exstrophy. He underwent first-stage cloacal exstrophy repair. The intraoperative findings included a bi-halved bladder and phallus, and the ureters were not appreciated, but there was continuous urine leakage from the bi-halved bladder and no uterus or ovaries. Poorly formed cecum, cecal-cutaneous fistula with an everted part of the terminal ileum protruding outside (mucosa-out), no transverse, no descending colon, collapsing small bowel, left undescended testis in the inguinal region, and right abdominal undescended testis. He first underwent surgery, which involved ileostomy, omphalocele closure and proper bladder exstrophy construction.

The second patient, a 6-day-old female, had a similar presentation and physical findings as the first patient did, except that she had elephantoid trunk deformity with a cecal fistula, bifid clitoris, two cervical orifices, and two uteri completely separated with ovaries. Mobilization of the hindgut, closure of the cecal fistula, and proper bladder exstrophy after repair of the posterior wall were performed.

The third patient was a 10-day-old female, similar to the second patient, but this patient presented with a left leg deformity with wide diastasis. In this case, the urinary bladder was not bivalved, and the cecal fistula had perforated just below the posterior wall of the urinary bladder. A mild omphalocele, bifid clitoris and vagina, one cervical orifice, and two uteri completely separated, with ovaries observed. Mobilization of the hindgut, closure of the cecal fistula, and proper bladder exstrophy after repair of the posterior wall were performed. The postoperative period was uneventful.

**Clinical discussion:**

Surgical management of cloacal exstrophy is typically undertaken in the newborn period (48–72 h) as a combined effort between pediatric surgery and urology. In the setting of associated spinal dysraphism, neurosurgical consultation and closure should be undertaken as soon as the infant becomes medically stable. Early operation minimizes bacterial colonization of exposed viscera and may decrease the need for pelvic osteotomy. The goals of treatment include securing the abdominal wall and bladder closure, preserving renal function, preventing short bowel syndrome, creating functional and cosmetically acceptable genitalia, and attaining acceptable urinary and fecal continence.

**Conclusion:**

Cloacal exstrophy remains a rare and complex congenital anomaly characterized by an array of anatomical defects affecting multiple organ systems. With respect to the approach of this congenital malformation, it is therefore important that these individuals and their families remain under the care of a multidisciplinary team of providers who can offer medical care, counseling and life-long follow-up.

## Highlights

• Cloacal exstrophy It is the most serious form of anomaly that is described pelvic malformations in a spectrum of anomalies known as exstrophy-epispadias complex (EEC).
• OEIS syndrome (Omphalocele–Exstrophy–Imperforate Anus–Spinal Anomalies Complex) is characterized by exstrophy of the urinary bladder and cecal plate through an abdominal wall defect, anal atresia, colonic hypoplasia, omphalocele, and anomalous genitalia.
• Bladder exstrophy, a classic bladder exstrophy, exposing the posterior part of bladder, and ureters outward.

## 1 Introduction and importance

Cloacal exstrophy (CE) is defined as a complex anomaly that affects the urogenital and intestinal tracts [1]. It is the most serious form of anomaly that is described within the term exstrophy-epispadias complex. These malformations usually pose a challenge in the management of particular conditions, as most of these forms require multiple surgeries with multidisciplinary approaches, including reconstructive urologists, pediatric surgeons, orthopedic surgeons, endocrinologists, pediatricians, psychologists and nutritionists [2]. Additionally, some patients present with ambiguous genitalia, which is another aspect that needs to be taken into consideration during the management of this condition [3].

The risk of recurrence of bladder exstrophy in a given family is approximately 1 in 100. The risk of bladder exstrophy in the offspring of individuals with bladder exstrophy and epispadias is 1 in 70 live births, a 500-fold greater incidence than in the general population [2,4]. A 10-fold increase in exstrophy births to mothers who had received large doses of progesterone in the early part of the first trimester, has been reported from other research [5].

A correct prenatal diagnosis of CE is rarely made. In a study reviewing antenatal sonographic findings in patients born with CE, anomalies were detected in 54 % of cases, but the correct diagnosis of CE was only 6 %. Most of these cases are misdiagnosed as hydronephrosis. Prenatal counseling is important to prepare parents to raise a child with a special trait [3]. Early prenatal diagnosis is crucial not only for early management but also for the long-term quality of life of CE patients [6].

## 2 Case presentation

**Fig 1:**
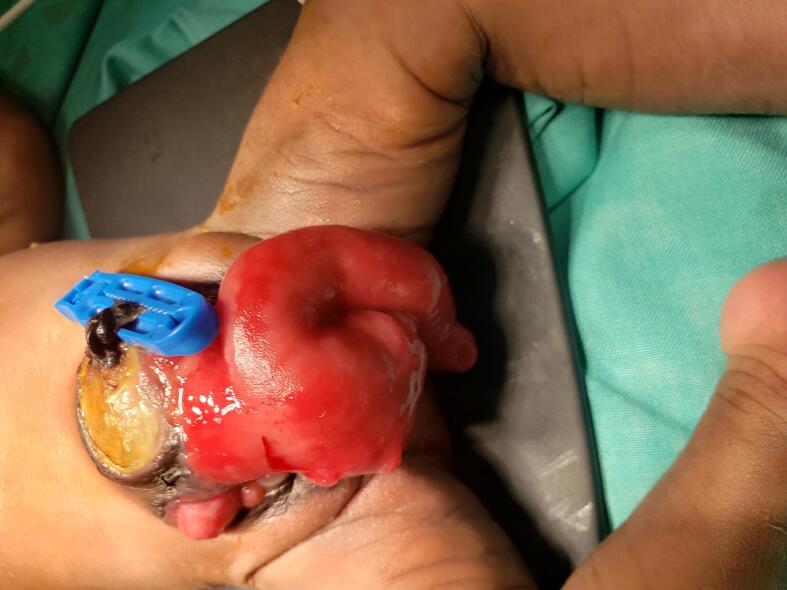
1st case shows a cloacal exstrophy.

**Fig. 2:**
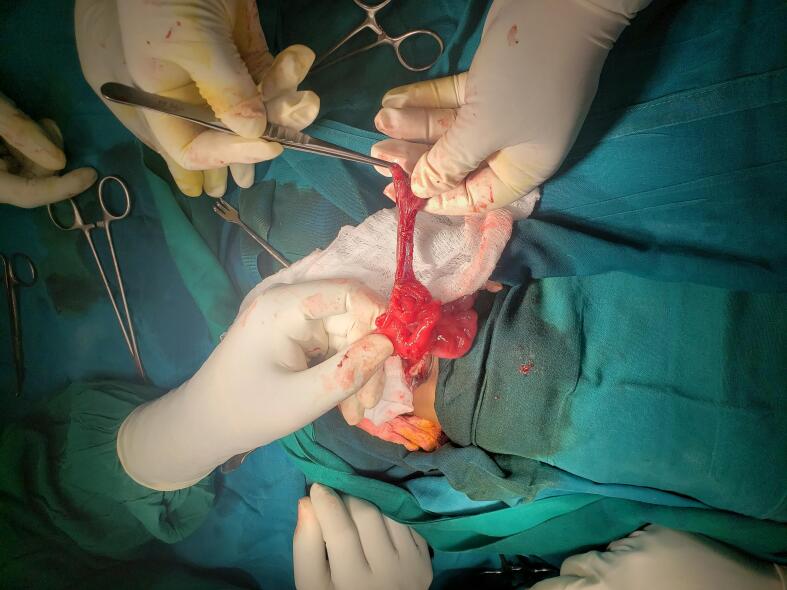
1st case shows intraoperative, blind loop end of the caecum.

**Fig. 3:**
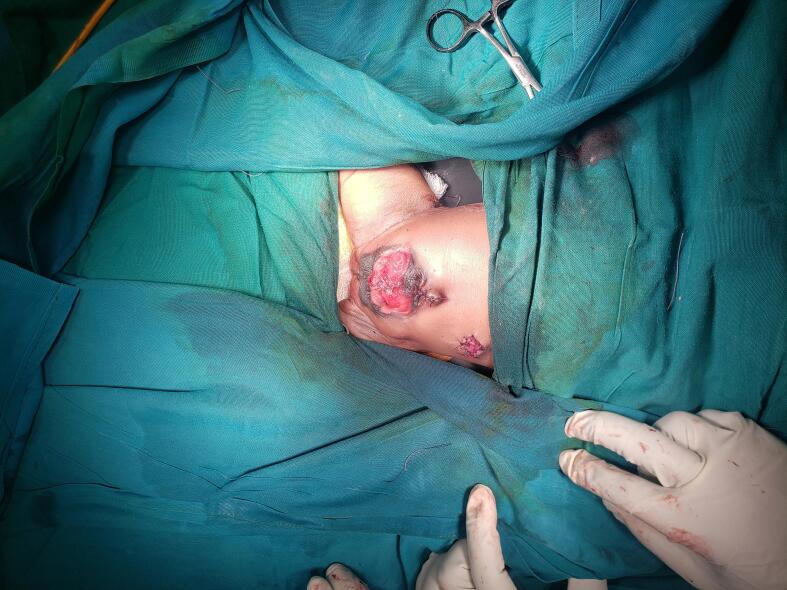
1st case, After creation of proper bladder extrophy and caecostomy.

The first patient, a baby who was 8 days of life and referred from a peripheral hospital, presented with classic features of cloaca exstrophy, the OEIS complex comprises a combination of defects including omphalocele, exstrophy of the cloaca, imperforate anus, and spinal defects. He underwent first-stage cloacal exstrophy repair by pediatric urologist and the team. The intraoperative findings included a bi-halved bladder and phallus, and the ureters were not appreciated, but there was continuous urine leakage from the bi-halved bladder and no uterus or ovaries. Poorly formed cecum, cecal-cutaneous fistula with an everted part of the terminal ileum protruding outside (mucosa-out), no transverse, no descending colon, collapsing small bowel, left undescended testis in the inguinal region, and right abdominal undescended testis. He first underwent surgery, which involved ileostomy, omphalocele closure and proper bladder exstrophy construction. He stayed in the ward for 11 days and was discharged waiting for the next surgery, which included bladder exstrophy closure, penile reconstruction and orchidopexy, Fig. 1, Fig. 2, Fig. 3.

**Fig. 4:**
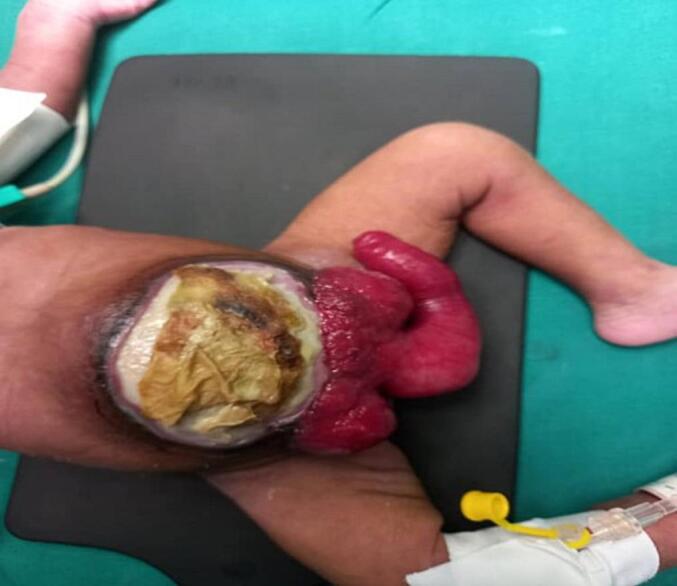
2nd case case shows a cloacal exstrophy with big omphalocele.

**Fig. 5:**
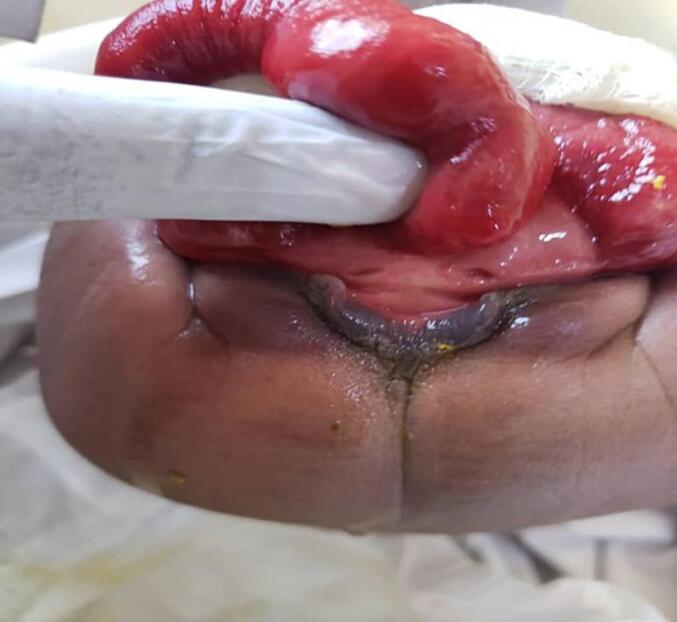
2nd case, cloacal exstrophy with ureteric orifice exposed.

**Fig. 6:**
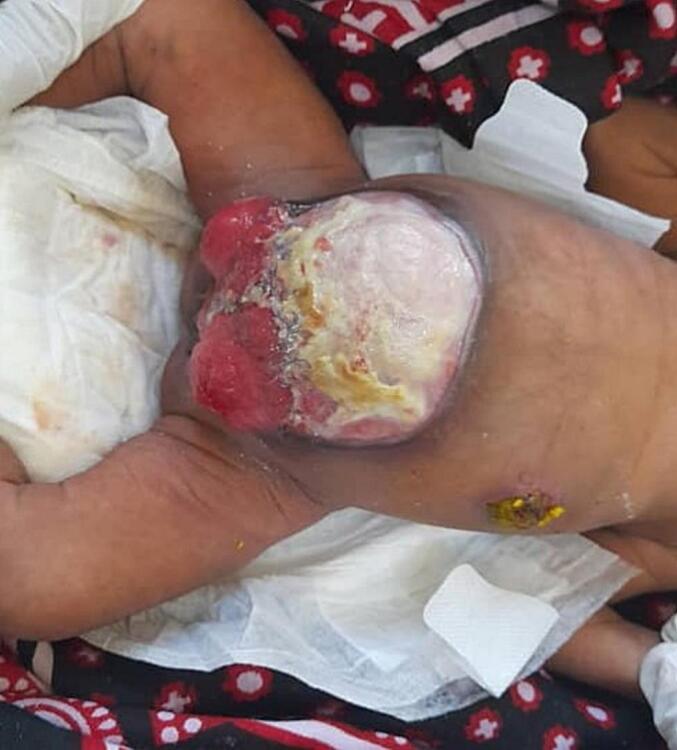
2nd case After creation of proper bladder extrophy and caecostomy, and omphalocele was left untouched.

The second patient had a similar presentation and physical findings as the first patient did, as for her elephantoid trunk deformity with a cecal fistula bifid clitoris, two cervical orifices, and two uteri completely separated with ovaries. Mobilization of the hindgut, closure of the cecal fistula, and proper bladder exstrophy after repair of the posterior wall were performed. It was difficult to repair the omphalocele because of an inadequate abdominal cavity. The baby is progressing well in the ward. This patient will require panel discussions with other specialties, such as gynecologists and pediatric surgeons, before reconstruction phase. Fig. 4, Fig. 5 Fig. 6.

**Fig. 7:**
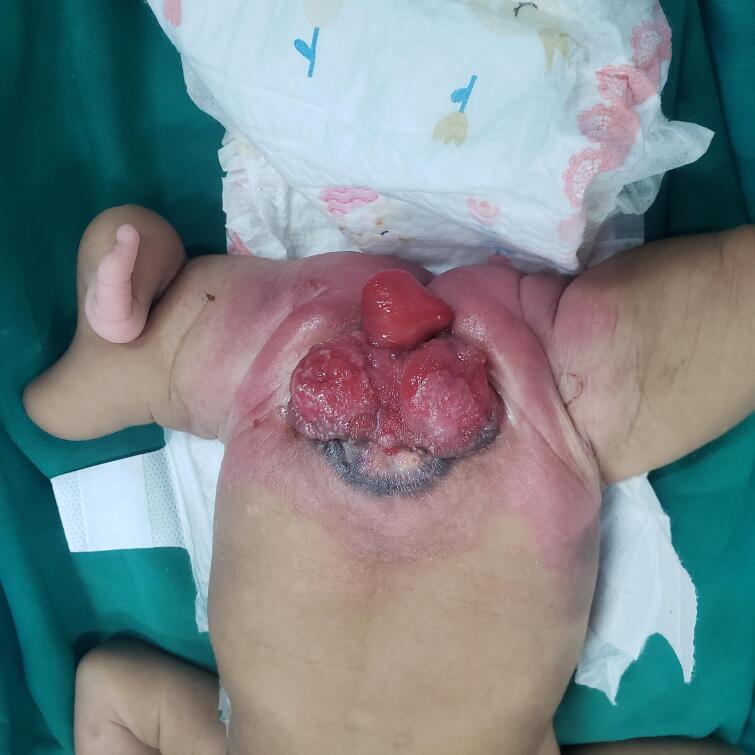
3rd case. A cloacal exstrophy female patient.

**Fig. 8:**
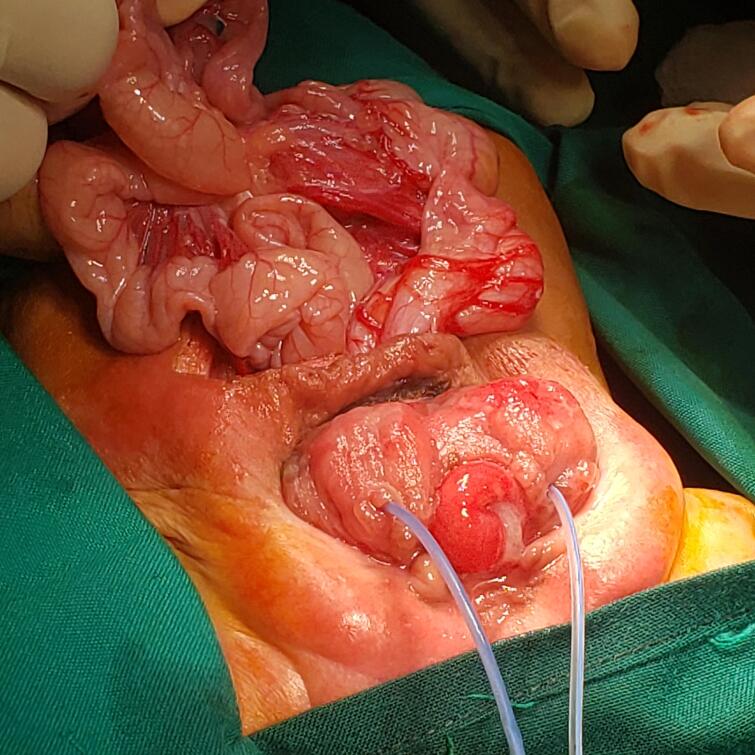
3rd case shows intraoperative, blind loop end of the caecum, catheterized ureteric orifice.

**Fig. 9:**
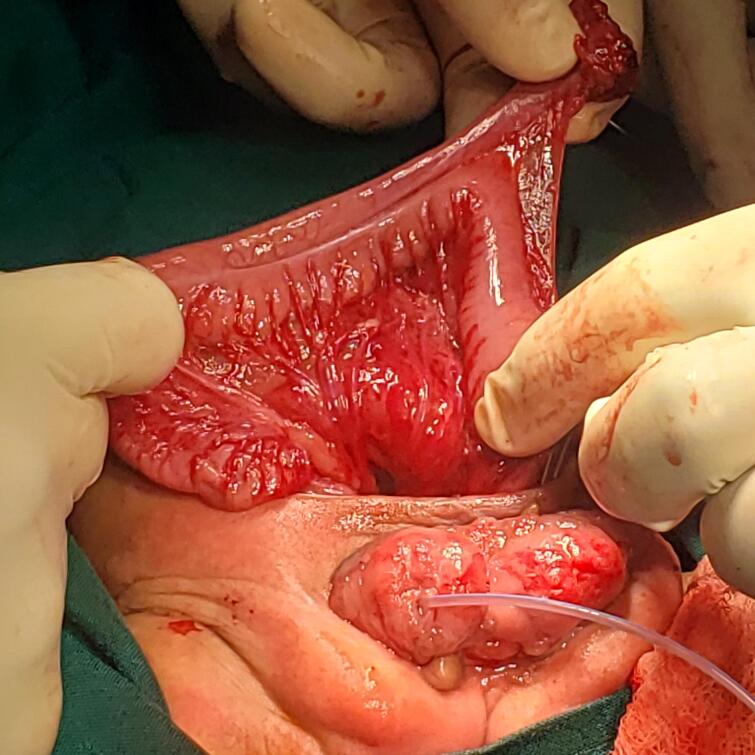
3rd case Fig. 8, 3rd case shows intraoperative, blind loop end of the caecum, catheterized ureteric orifice.

**Fig. 10:**
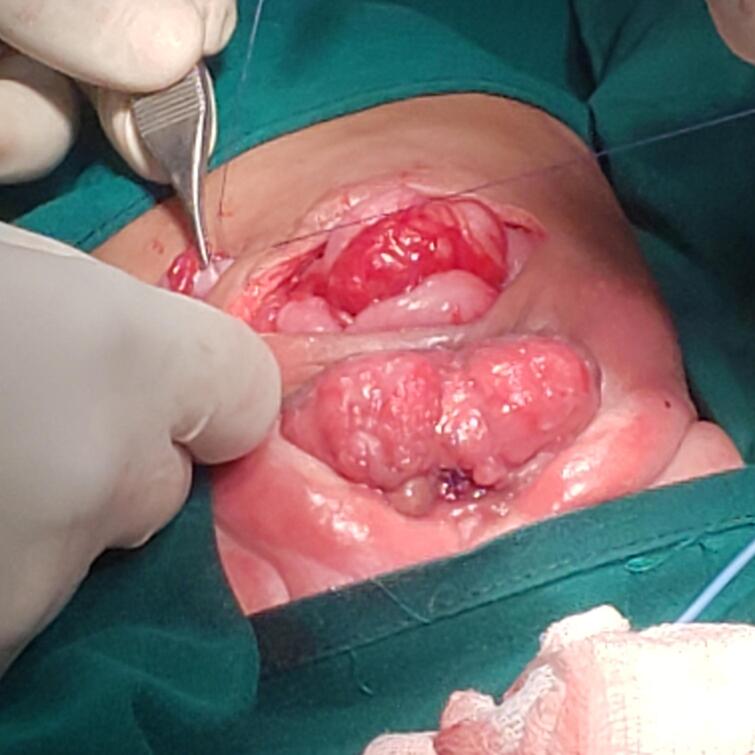
3rd case reconstruction to a proper extrophy.

The third patient was a 10-day-old female, similar to the second patient, but this patient presented with a left leg deformity with wide diastasis. In this case, the urinary bladder was not bivalved, and the cecal fistula had perforated just below the posterior wall of the urinary bladder. A mild omphalocele, bifid clitoris and vagina, one cervical orifice, and two uteri completely separated, with ovaries observed. Mobilization of the hindgut, closure of the cecal fistula, and proper bladder exstrophy after repair of the posterior wall were performed. The postoperative period was uneventful, Fig. 7, Fig. 8, Fig. 9, Fig. 10.

## 3 Clinical discussion

Immediately after birth and stabilization of the newborn, exposed organs/mucosal surfaces should be protected by enclosing the infant's lower torso in a bowel bag or by first moistening surfaces with saline and covering them with sterile plastic wrapping.

The urologic exam should attempt to determine genetic sex, the size of hemibladders, and the presence of spinal dysraphism [7]. Baseline renal function and electrolyte and hematologic status should be determined through routine serologic testing. Karyotyping can be performed if gender has not been previously determined or is not obvious on exam. Initial imaging should include plain films of the chest and spine along with head, abdominal, renal, and spinal ultrasounds.

Different approach has been proposed in management, one-stage approach versus a staged approach, and whether a potential need for osteotomy, and what type of osteotomy needed. Modern approaches and techniques taken in the staged reconstructive approach will be more discussed in with our cases [8,9].

Surgical management of cloacal exstrophy is typically undertaken in the newborn period (48–72 h) as a combined effort between pediatric surgery and pediatric urology. In the setting of associated spinal dysraphism, neurosurgical consultation and closure should be undertaken as soon as the infant becomes medically stable. Early operation minimizes bacterial colonization of exposed viscera and may decrease the need for pelvic osteotomy [10]. The goals of treatment include secure abdominal wall and bladder closure, preservation of renal function, prevention of short bowel syndrome, creation of functional and cosmetically acceptable genitalia, and attainment of acceptable urinary and fecal continence [11].

First staged Modern Closure, The first steps of reconstruction i) separation of the cecal plate, ii) rescue of the hindgut, iii) Tubularization of the cecum, iv) re-approximation of the bladder's halves into classical exstrophy, v) omphalocele closure versus delayed closure, and vi) creation of an end colostomy [8]. Although, all approaches emphasize initial separation of the intervening cecal plate from the two bladder halves, omphalocele closure, and hindgut preservation. Currently, after tabularization of the exstrophied cecum, it is recommended that the hindgut segment be removed as an end ileostomy. This hindgut segment may be anastomosed in an isoperistaltic or retro peristaltic fashion to decrease motility and generate formed stool. In rare instances, when the hindgut remnant is not utilized, it may be left as a mucous fistula for use in subsequent urologic or vaginal reconstruction. The omphalocele is excised to facilitate abdominal wall closure. Closure may be complicated by the development of abdominal compartment syndrome, and in cases of large omphaloceles, a complete initial reduction may not be possible. In this setting, a silo device may be utilized. Alternatively, the omphalocele may be allowed to re-epithelialize, thus converting it to a ventral hernia, which may be repaired at a later time. The hemibladders are then dissected freely and approximated in the posterior midline. In infants with few other associated malformations who are medically stable, complete closure of the abdominal wall, bladder, and phallic halves may be undertaken at this point in a single-stage procedure with or without osteotomy [12].

Second staged Modern Closure (6 Months–2 Years of Age). The goals of the second stage of reconstruction include i) bladder closure, ii) abdominal wall closure, and iii) reconstruction of external genitalia in select patients. Pelvic osteotomy is highly recommended as part of closure to decrease abdominal wall tension following pubic bone approximation. It also increases the chance of a successful bladder closure and decreases risk of wound complications, like bladder dehiscence, penile ischemia, urethra-cutaneous or Vesico-cutaneous fistula, bladder outlet obstruction, urinary tract infection, compromised renal function, and prolonged ileus [8,13].

Third staged Modern closure of reconstructions (4 - 6 years of age) is i) bladder neck reconstruction, ii) augmentation cystoplasty to increase bladder capacity, iii) correction of vesicoureteral reflux iv) creation of a catheterizable channel for timed bladder emptying. At this stage, patient and family members or caregivers need to be socially accepting, adherence to clean intermittent catheterization to avoid other complication such as renal injury from reflux, urosepsis due to prolong urine stasis, increase upper tract pressure, bowel perforation [8]. Intraoperative findings in all case, no adhesion, since both cases were fresh case surgery, however the aim was to create a proper exstrophy, took an average of 2 h per case, with minimal blood loss less than 100mls. Basically, wound care, specifically stoma care, and apparently, there was no complication, both cases fared well, they are waiting for the next plan of reconstruction of bladder exstrophy, and bladder neck reconstruction, anoplasty. Three cases were followed up to 6-month post-surgery, they are waiting for second stage.

## 4 Conclusion

Cloacal exstrophy remains a rare and complex congenital anomaly characterized by an array of anatomical defects affecting multiple organ systems. As evident from this review, the optimal treatment of cloacal exstrophy must address many different aspects of an individual, from the timing and type of repair to genital reconstruction and quality of life issues. Advances in medical and surgical management have allowed for dramatically improved survival and continence rates; however, the chronic nature of cloacal exstrophy must be emphasized. Afflicted patients generally require multiple surgical procedures and face medical, psychological and social challenges throughout their lives. It is therefore important that these individuals and their families remain under the care of a multidisciplinary team of providers who can offer medical care, counseling and life-long follow-up.

## 5 Study design

A retrospective, single-centre, non-consecutive case series.

## 6 Setting

Setting was a single centre, Muhimbili national hospital, Tanzania.

## 7 Method

The work has been reported in line with the process checklist criteria [14].

## Consent

Written informed consent was obtained from the patient's guardians for publication of this case series report and accompanying images. Copies of the written consents are available for review by the Editor-in-Chief of this journal on request.

## Ethical approval

The present study was exempted by the Ethics Committee of The Affiliated Hospital of Muhimbili national hospital, Tanzania, as this paper reports a case that emerged during normal surgical practice.

## Funding

No funding concerning this article.

## Author contribution

Geofrey Chiloleti – Assistant Surgeon, Study concept & writing Manuscript.

Gabriel Mtaturu – Chief Surgeon, Correction manuscript.

Sirili Harya Chief surgeon.

Herry Kibona Assistant surgeon.

Fransia Mushi –Supervisor.

Boniface Kilangi-Pre, Post operative care and follow up.

## Guarantor

Gabriel Mtaturu.

Sirili Harya.

Herry G Kibona.

## Research registration number

1. Name of the registry: NIL.
2. Unique identifying number or registration ID: NIL.
3. Hyperlink to your specific registration (must be publicly accessible and will be checked).

## Conflict of interest statement

The authors declare that they have no competing interests.
